# Eye contact effects on social preference and face recognition in normal ageing and in Alzheimer’s disease

**DOI:** 10.1007/s00426-017-0955-6

**Published:** 2017-12-01

**Authors:** D. Lopis, M. Baltazar, N. Geronikola, V. Beaucousin, L. Conty

**Affiliations:** 1 Laboratory of Human and Artificial Cognition (CHArt), UPL, Univ Paris Nanterre, 92000 Nanterre, France; 2 Athens Association of Alzheimer’s Disease and Related Disorders, Athens Day Care Center, Athens, Greece; 3 Normandie Univ, UNIROUEN, CRFDP, 76000 Rouen, France

## Abstract

Perceiving a direct gaze (i.e. another individual’s gaze directed to the observer leading to eye contact) influences positively a wide range of cognitive processes. In particular, direct gaze perception is known to stimulate memory for other’s faces and to increase their likeability. Alzheimer’s disease (AD) results in social withdrawal and cognitive decline. However, patients show preserved eye contact behaviours until the middle stage of the disease. The eye contact effects could be preserved in AD and be used to compensate for cognitive and social deficits. Yet, it is unknown whether these effects are preserved in normal ageing. The aim of this study was to address whether the positive effects of eye contact on memory for faces and likeability of others are preserved in healthy older adults and in patients with early to mild AD. Nineteen AD patients, 20 older adults and 20 young adults participated in our study. Participants were first presented with faces displaying either direct or averted gaze and rated each face’s degree of likeability. They were then asked to identify the faces they had previously seen during a surprise recognition test. Results showed that the effect of eye contact on other’s likeability was preserved in normal ageing and in AD. By contrast, an effect of eye contact on memory for faces seems to emerge only in young participants, suggesting that this effect declines with ageing. Interestingly, however, AD patients show a positive correlation between ratings of likeability and recognition scores, suggesting that they implicitly allocated their encoding resources to most likeable faces. These results open a new way for a “compensating” therapy in AD.

## Introduction

The eyes are one of the first visual social cues processed by human infants and it is probably the most important throughout normal postnatal development and lifespan (Farroni, Johnson, & Csibra, 2004; Farroni, Csibra, Simion, & Johnson, 2002). Gaze is an essential social signal playing a role in the regulation of inter-individual exchanges (Kleinke, 1986). Among all gaze directions, direct gaze, i.e. another individual’s gaze directed to the observer that leads to eye contact, has raised a particular interest among researchers in social sciences over the last decades. In most species, the perception of direct gaze evokes an aversive response, probably because it is a salient signal of potential threat (Emery, 2000). In humans, by contrast, direct gaze has the power to implicitly influence a wide range of cognitive processes and behaviours in a way that generally favors social interactions (see Senju & Johnson, 2009; Conty, George, & Hietanen, 2016) at least when the exposure to direct gaze does not exceed 3 or 4 s (Argyle & Cook, 1976; Argyle & Ingham, 1972; Binetti, Harrison, Coutrot, Johnston, & Mareschal, 2016).

Direct gaze has been shown to capture attention and to receive prioritized visual processing (Böckler, van der Wel, & Welsh, 2014; Conty, N’Diaye, Tijus, & George, 2007; Senju & Hasegawa, 2005; Conty et al., 2010), as soon as the early stages of human life (Farroni et al., 2002). The finding that attentional capture by direct gaze seems functional from birth, suggests that it is triggered by low-level visual features (Ando, 2002; Kobayashi & Kohshima, 2001; Langton, 2000). However, direct gaze perception has further effects on human cognition. The eye contact effects have recently been classified in four main categories (Conty et al., 2016): eye contact leads to general positive appraisals of others (Kleinke, 1986; Kuzmanovic et al., 2009), activates pro-social behaviours (Baillon, Selim, & van Dolder, 2013; Fathi, Bateson, & Nettle, 2014; Nettle, Nott, & Bateson 2012), improves memory for other’s identity and discourses (Conty & Grèzes, 2011; Hood, Macrae, Cole-Davies, & Dias, 2003; Vuilleumier George, Lister, Armony, & Driver, 2005) and enhances several forms of self-awareness (Baltazar et al., 2014; Pönkänen, Peltola, & Hietanen, 2011).

It has been emphasized that the eye contact effects mainly reflect a positive impact on human cognition and, for this reason, could have a therapeutic potential (Conty et al., 2016; Baltazar & Conty, 2016). Eye contact effects may be used not only to preserve the quality of social interaction (by promoting intentions to communicate, positive evaluations of others and pro-social actions), but also to prevent from cognitive decline (especially by stimulating self-awareness and memory), at least in patients with preserved direct gaze processing. To this respect, patients suffering from Alzheimer’s disease (AD) could be a relevant clinical population so as to investigate the eye contact effects. To the best of our knowledge, however, no studies to date have been conducted to investigate the preservation of the eye contact effects in AD, or even to study their evolution in normal ageing.

AD results not only to memory impairment. It is also characterized by psycho-behavioural anomalies and language disabilities that necessarily appear at some point during the disease’s evolution and impoverish the patient’s relations with others, even at the early stages of the disease (Parsons-suhl, Johnson, Mccann, & Solberg, 2008; Rousseaux, Sève, Vallet, Pasquier, & Mackowiak-Cordoliani, 2010; Snyder, 2001). This contributes to patients’ social withdrawal, which increases with their progressive cognitive decline, and their lack of awareness of these difficulties (Clare & Woods, 2008). Interestingly, however, the processing of eye direction as well as eye contact behaviour seems to be preserved in patients with early and moderate AD. Sturm et al. (2011) recently demonstrated that the frequency of eye contact and the physiological reactivity to this stimulus are preserved in the early stage of the disease. Another study has shown that patients in mild or moderate stage of the disease were impaired in facial expression recognition but not in gaze direction determination, in contrast to Fronto-Temporal Dementia patients who were impaired in both tasks (Bediou et al., 2009 but see Insch, Slessor, Warrington, & Phillips,^2016^).

In normal ageing, evidences indicate that older adults attend less to the eye region of emotional faces than young people (Circelli, Clark, & Cronin-Golomb, 2013; Murphy & Isaacowitz, 2010; Sullivan, Ruffman, & Hutton, 2007). They are also less willing to engage joint attention compared to younger adults (i.e. they have reduced tendency to follow other’s gaze direction toward the surrounding space). However, this age difference is unlikely to be related to the difficulty in detecting gaze patterns since older adults had intact ability to detect both direct gaze and clearly averted gaze [i.e. gaze averted by at least 0.38° to the left or right (Slessor, Phillips, & Bull, 2008)].

It is also established that both normal ageing and AD pathology affect capacities for mentalizing, although with a different magnitude (see Kemp, Després, Sellal, & Dufour, 2012 for a review). In parallel, recent evidences show that a basic form of mentalizing process (leading to the belief of being watched) is often recruited during eye gaze processing and it is likely to play a central role in the “Watching eyes” effects (Conty et al., 2016; Hamilton, 2015). However, it has been advanced that the decline of mentalizing abilities in typical ageing and in AD follows, in a reverse order, the developmental stages of early life (Castelli et al., 2011; Moran, 2013). Since the eye contact effects appear very early in human development (Conty et al., 2016; Conty and George, 2008), one may expect that they are preserved until the latest stage of normal ageing and of AD.

The aim of our study was to address the question of the persistence of “Watching Eyes” effects in healthy older adults and in AD patients. We focused on the impact of direct gaze on the modulation of others’ appraisal and on memory for faces for two main reasons: First, the eye contact effects have proven to be robust, and second, they can be easily evidenced with a simple experimental procedure (e.g. Conty & Grèzes, 2011; Mason, Hood, & Macrae, 2004; Wirth, Sacco, Hugenberg, & Williams, 2010). We exposed participants to digital pictures of unfamiliar faces with different eye directions (direct vs. averted), and for each face, we asked them to rate how likeable each individual was. This measure allowed us to test the effect of direct gaze on other’s appraisal. After an interfering task, participants were submitted to a surprise forced-choice recognition task on the faces, allowing us to test for an effect of direct gaze on face memory. We predicted that the faces initially displayed with a direct gaze would be judged more likeable and would be better recognized compared to the faces initially gazing away from the participant. We recruited AD patients, matched older participants without cognitive impairment and healthy young subjects, to distinguish effects pertaining to normal or pathological ageing. We hypothesized that direct gaze effects would be observed in all groups. Furthermore, since it has been shown that likeability judgements may play a mediating role in face recognition (Bruce & McDonald, 1993; Mueller, Heesacker, & Ross, 1984), we also explored the correlation between recognition rate and likeability judgement in each group of participants.

## Method

### Participants

A total of 59, right-handed, native French-speaking participants were included in the study: nineteen patients with a diagnosed AD (13 women; mean age ± standard deviation = 81.2 ± 4.9 years), 20 community-dwelling healthy older participants (16 women; mean age = 78.3 ± 5.7 years) and 20 healthy young participants (12 women; mean age = 23.7 ± 3.6 years). Young participants were recruited by advertisements spread on a French internet database of volunteers willing to participate in psychology or neuroscience research. Older participants were community dwelling and were recruited by advertisements and notices distributed through senior citizen organizations in the Paris areas. The patients with AD were recruited from a local memory center and were at the early to mild stage of the disease (MMSE between 19 and 24; Feldman & Woodward, 2005). All participants had normal or corrected-to-normal vision and were naive to the aim of the experiment. They provided written informed consent according to institutional guidelines of the local research ethics committee (who stated on the compliance with the Declaration of Helsinki). The whole procedure was approved by the local ethics committee (CPP n 2014-A01141-46, 2015/07/08).

All participants underwent structured interviews and neuropsychological testing to assess cognitive functioning. A full description of the groups of participants is presented in Table 1. The diagnosis of probable or possible AD was assigned to patients by a neurologist according to the criteria of the National Institute of Neurological and Communicative Disorders and Stroke and the Alzheimer’s disease and Related Disorders Associations (NINCDS/ADRDA; McKhann et al., 2011). AD patients were excluded if they were judged to be unable to understand task instructions. None of the AD patients was reported to have prosopagnosia. For controls, the following exclusion criteria were applied: history of neurological disorders, traumatic brain injury with loss of consciousness and significant history of psychological or psychiatric disorders.

**Table 1:** Means and SDs of Demographics, General Neuropsychological Efficiency and Depression scores

	Young adults	Older adults	AD patients	Differences between young adults and older adults		Differences between older adults and AD patients	
				*t* value	*p* value	*t* value	*p* value
*N* (F:M)	20 (12:8)	20 (16:4)	19 (13:6)	–^a^	n.s	–^a^	n.s
Age (years)	23.7 (3.6)	78.3 (5.7)	81.2 (4.9)	− 35.9	< 0.000	− 1 .6	n.s
Level of education (years)	14.9 (1.2)	10.2 (3.5)	9.5 (4.3)	5.5	< 0.000	0.4	n.s
General cognitive efficiency MMSE (30)^b^	29.2 (1.1)	27.9 (1.3)	23 (2.8)	3.1	< 0.01	6.9	< 0.000
Frontal efficiency FAB (18)^b^	17.4 (0.8)	15.6 (1.6)	14.5 (1.8)	4.2	< 0.000	1.9	n.s
Episodic memory 5-words test (10)^b^	10.0 (0.0)	9.9 (0.4)	6.1 (2.1)	1.0	n.s	7.7	n.s
Attention and working memory							
Forward digit span	6.9 (1.2)	4.9 (1.1)	5.4 (0.9)	5.1	< 0.000	− 1.5	n.s
Backward digit span	4.9 (1.2)	3.8 (0.7)	3.8 (0.9)	3.2	< 0.01	− 0.1	n.s
Verbal fluency(*P*)	28.9 (5.7)	18.1 (4.7)	13.9 (6.2)	6.2	< 0.000	2.3	< 0.05
Depression GDS (cut-off < 7/15)	–^c^	1.9 (1.3)	2.2 (1.3)	–^c^	–^c^	0.6	n.s

Two-tailed *t* tests for independent samples were used.

*SD* standard deviation, *n.s*. not significant, *AD* Alzheimer’s disease, *MMSE* mini mental state examination; *FAB* frontal assessment battery, *GDS* geriatric depression scale

^a^ Gender distribution across groups was tested using a two-sided Fisher exact test for count data

^b^ Maximum possible score

^c^ Not applicable. Young adults were screened for present major depression using the mini international psychiatric interview 5.0.0 (French version, Lecrubier et al., 1998)

The neuropsychological evaluation consisted in exploring global cognition with the Mini Mental State Examination (MMSE; Folstein, Folstein, & McHugh, 1975), frontal lobe and executive functions with the Frontal Assessment Battery (Dubois, Slachevsky, Litvan, & Pillon, 2000), productive language with a verbal fluency task (Cardebat, Doyon, Puel, Goulet, & Joanette, 1990), episodic memory with the 5-words test (Dubois et al., 2002), attention and working memory with the forward and backward digit spans (Wechsler, 1997). Regarding psychiatric evaluation, older participants (healthy controls and AD patients) fulfilled the 15-items Geriatric Depression Scale (Yesavage et al., 1983) and those who scored 7 or more on this scale were excluded from the study. The Mini International Psychiatric Interview 5.0.0 (French version, Lecrubier, Sheehan, Hergueta, & Weiller, 1998) was administered to young participants to screen for present major depression.

All healthy participants had performances within the normal range in all neuropsychological screening tests (i.e. having a score no more different than 1.65 *SD* compared to the mean of their group of reference, as provided in the norms of each test). All healthy older adults had an MMS score superior or equal to 26. None of them expressed any complaints about their memory. All were paid for their participation.

Healthy older adults and AD groups were matched for age, gender distribution across groups, years of education and level of depression (see details in Table 1).

### Stimuli

Stimuli consisted of 120 static greyscale photographs of 40 individuals (20 men/20 women) which were selected from a database of digitized portraits of young adult faces (see Conty, N’Diaye, Tijus, & George, 2007; Vuilleumier, George, Lister, Armony, & Driver, 2005). All faces had neutral expression and involved individuals unknown to our participants. The age of each individual ranged from 20 to 60 years and our stimuli selection included approximately 1/3 of young-looking faces, 1/3 of middle-aged-looking faces and 1/3 of old-looking faces. Head direction was always oriented straight toward the observer. Each individual was presented in three different views: one with the eyes directed straight toward the observer (Direct Gaze condition), one with the eyes averted by 30° toward the right side from the observer position (Averted Gaze condition) and one with closed eyes. Face stimuli with averted gaze were mirrored to obtain both left-averted and right-averted gaze pictures.

### Procedure

Participants sat approximately at 70 cm in front of a Dell computer with a 15.6 inches screen (with a resolution of 1366 × 768 pixels) on which stimuli were shown on a black background. E-Prime® 2.0 software was used to control stimulus presentation, response recording and latency (Psychology Software Tools, 2002).

The experiment was divided into three parts: an initial encoding phase, a 5-min interfering task and a surprise recognition task. Two groups of 20 faces: A and B were created, each composed of 20 different individuals (10 men/10 women): when stimuli from group A were used during the encoding phase, stimuli from group B constituted distractors in the subsequent surprise recognition task. This experimental sequence was used for half of the participants for each group and the other half was submitted to the reverse sequence.

**Fig. 1:**
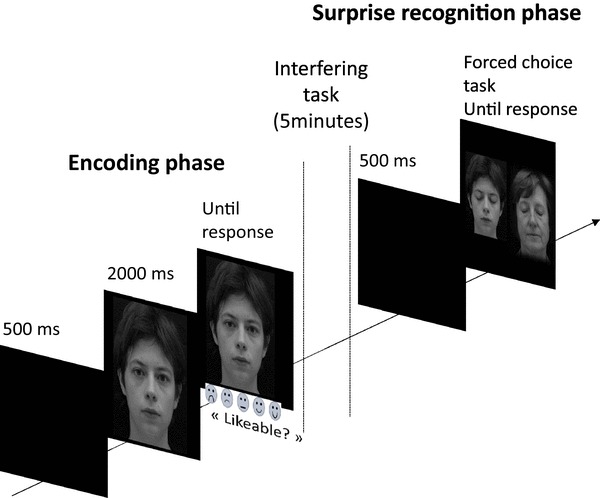
Illustration of the experimental design. During the encoding phase, participants were presented one by one with 20 faces displaying either direct or averted gaze and were asked to rate each face’s degree of likeability on a Likert scale ranged from 1 (“Not at all likeable”) to 5 (“Very likeable”). After a 5-min interfering task, they were shown 20 pairs of faces with closed eyes and were asked, at each trial, to report which of the two faces they have seen before (surprise memory phase)

During the initial encoding phase, participants were presented with 20 different faces (from group A or B), one by one, in a randomized order. The association between face’s identity and gaze direction was also randomized across participants, with the constraint that five men and five women were shown with a direct gaze and the other five men and five women were shown with averted gaze (right-averted for half of the participants and left-averted for the other half). For each face, participants were asked to rate the face’s degree of likeability on a Likert scale ranged from 1 (“Not at all likeable”) to 5 (“Very likeable”). A wooden cover, placed on the computer keyboard, allowed the participants to use only five keys to enter their response. Stickers displaying different degrees of smiling symbolic faces (representing the degrees of likeability) were placed on the keys to provide a visual aid (see Fig. 1).

Each trial started with a 500 ms presentation of a fixation cross (visual angle of 2°×°2) which was located at the level of the to-be-presented face’s eyes. Then the face appeared on the screen, covering a visual angle of approximately 13° horizontally and 16° vertically. After 2000 ms, the word “Likeable?” appeared under the face as a prompt for the participants to enter their response. Participants could not enter a response before the appearance on the screen of the word “Likeable?”, so that each individual’s face was seen during at least 2000 ms. Each face remained on the screen until a response was given. Immediately after the participant’s response, a black screen was displayed during 1000 ms, preceding the fixation cross of the next trial.

Immediately after the encoding phase, a 5-min interfering phase followed. During this phase, participants were submitted to a verbal fluency task (see Sect. "Participants"). They were first asked to evoke as many words beginning with the “P” letter as possible, and then to do the same for words belonging to the category of “Animals”.

This interfering phase was directly followed by a surprise recognition test in which participants were shown 20 pairs of faces with closed eyes (see Smith, Hood, & Hector, 2006 for similar procedure). Each trial started with the presentation of a fixation cross during 500 ms at the center of the screen. Then, two same-sex individual’s faces appeared simultaneously on the screen, side by side, one on the left visual field, the other one on the right. Each face covered a visual angle of approximately 11° horizontally and 12° vertically. One of them had already been seen by the participant during the encoding phase (i.e. the OLD face), the other had not (i.e. the NEW face). Participants were asked to choose between the two faces the one they thought they had seen before using a two-choice button press. Participants were invited to press the left-key of the touchpad to select the face displayed on the left side of the screen and the right-key to choose the one displayed on the right. Each pair of faces remained on the screen until a response was given. The association between the OLD face and the NEW face was randomized across participants. The side of the screen in which the old face appeared was random, with the constraint that half of the OLD faces appeared on the left, and the other half appeared on the right for every participant. The presentation order of the OLD face was random. Faces were presented with closed eyes to specifically test the recognition of the identity of the face and to prevent participants from doing a superficial picture-matching task (Bruce, 1982).

At the end of the experiment, participants were also submitted to a debriefing interview. They were asked whether any of the faces used in the experiment was previously known to them and if they anticipated the incoming surprise recognition task during the likeability rating phase. All participants confirmed that none of the faces was familiar prior to testing. None of them anticipated the subsequent recognition task.

### Analysis

We conducted 2 × 3 two-way repeated measures ANOVAs with Gaze Direction (direct/averted) as within-subjects factor and Group (AD patients/older adults/young adults) as between-subjects factor on the following variables of interest: mean ratings of likeability in the encoding phase, mean time of exposure to the faces in the encoding phase (TEx, i.e. response time to the likeability judgement task + 2000 ms corresponding to the time during which the face was presented without the prompt), percentage of correct recognitions (Hits) and response times for correct answers (RT) in the surprise recognition test. Partial Eta-squared (*η*^2^_*p*_) are reported as effect size indexes. As suggested by Cohen (1988), we considered effect sizes as being small for *η*^*2*^_*p*_ < 0.06, medium for 0.06 ≤ *η*^2^ < 0.14, and marked for *η*^2^ ≥ 0.14. Planned comparisons were performed using bilateral Student *t* test when main effects or interactions were observed. For significant comparisons, Cohen’s d was used to determine effect size with *d* < 0.3 corresponding to a small effect, 0.3 < *d*  < 0.8 to a medium effect and *d*  > 0.8 to a large effect (Cohen, 1988).

We also performed across-stimuli correlations between mean ratings of Likeability and Recognition rates, within group. For each group of participants, we correlated the mean score of likeability and the mean recognition percentage obtained for each face independently of its gaze direction at the encoding phase. As three correlations were computed, the threshold of significance was set at *p* < 0.016. For each group, we provide the Pearson R and bilateral *p* values. Using the Fisher* r*–*z* transformation, we then assessed the significance of the difference between each pair of correlation coefficients. For each comparison, we provide the two-tailed *p* values.

In the group of older adults, the mean recognition rate of one face was under chance level (*p* = 0.05). This was also the case for one face in the group of AD patients. These faces were excluded from the analysis for their related group.

## Results

### Encoding phase

**Fig. 2:**
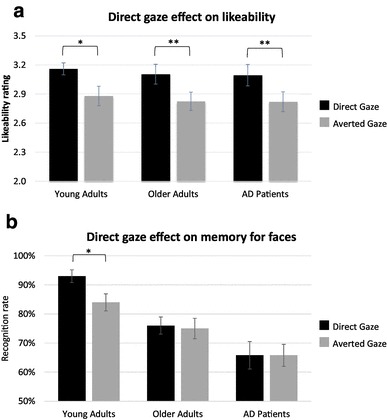
Behavioural results. **a** Ratings of likeability (mean and standard error to the mean) for faces presented with direct and averted gaze in young adults, healthy older adults and AD patients. **b** Percentage of recognition (mean and standard error to the mean) for faces presented with direct and averted gaze in young adults, healthy older adults and AD patients. ^*^p <  0.05. **p < 0.01

The 2 × 3 two-way repeated measures ANOVA with Gaze Direction as within-subjects factor and Group as between-subjects factor performed on the mean rate of likeability revealed a main effect of Gaze Direction (*F*_(1,56)_  = 22.8, *p* < 0.0001; *η*^*2*^_*p*_ = 0.28). Overall, during the encoding phase, participants rated faces with direct gaze as more likeable than faces with averted gaze (mean = 3.12 ± 0.40 vs. 2.84 ± 0.42). The interaction between Gaze Direction and Group was not significant (*F*_(2,56)_ = 0.001; *p* > 0.1), suggesting that this effect was of a comparable magnitude for each group. Planned comparison confirmed that the effect of Gaze Direction on likeability judgement was significant in all three groups of participants (AD patients: *t*_(18)_ =  2.92; *p* = 0.009, *d* = 0.67; healthy older adults *t*_(19)_ = 3.11; *p* = 0.006, *d* = 0.69; young adults *t*_(19)_ = 2.41; *p* = 0.02, *d* = 0.55*)* (Fig. 2a). There was no overall group effect on likeability ratings (*F*_(2,56)_ = 0.17; *p* > 0.1).

The 2 × 3 two-way repeated measures ANOVA with Gaze Direction as within-subjects factor and Group as between-subjects factor performed on the time of exposure to the face (TEx, i.e. 2000 ms + response time, see Methods) revealed a main effect of Group (*F*_(2,56)_ = 11.67; *p* < 0.0001; *η*^*2*^_*p*_ = 0.29). Planned comparisons showed that, overall, AD patients were slower in performing the likeability task than healthy older participants (respectively, mean = 6319 ± 3625 vs. 3988 ± 1363 ms, *t*_(37)_ = 2.68; *p* < 0.01, *d* = 0.93), who were slower than young adults (mean = 2959 ± 433, *t*_(38)_ = 3.21; *p* < 0.01, *d* = 1.14) (Table 2b). Crucially, no effect of Gaze direction, or interaction between Gaze Direction and Group, was found on this variable (all *p*_s_ > 0.1), indicating that all participants were exposed during the same amount of time to faces with direct and averted gaze (Table 2a).

**Table 2:** Means, SDs and *p* values of the time of exposure to faces in the encoding phase (TEx, i.e. 2000 ms of simple exposition to the face + response times to the likeability judgement task) and response times for correct answers in the surprise recognition test (TR for Hits) of AD patients, older adults and young adults, (a) for each experimental condition (direct/averted gaze direction) and (b) averaged across conditions

(a) For each experimental condition (direct/averted gaze direction)	TEx			TR for hits		
	Direct gaze	Averted gaze	*p* value (*t* test)	Direct gaze	Averted gaze	*p* value (*t* test)
AD patients (*n* = 20)	6287 ± 3823	6350 ± 3596	n.s	6079 ± 2631	6083 ± 3340	n.s
Older adults (*n* = 20)	3979 ± 1584	3997 ± 1311	n.s	3676 ± 1279	3700 ± 1173	n.s
Young adults (*n* = 19)	2900 ± 363	3018 ± 584	n.s	2765 ± 3091	2058 ± 607	n.s

(b) Averaged across conditions	Young adults (*n* = 20)	Older adults (*n* = 20)	AD patients (*n* = 19)	ANOVA	Differences between groups in planned comparisons	
TEx	2959 ± 433	3988 ± 1363	6319 ± 3625	*F* (2, 56) = 11.6***	A** B**	
RT for Hits	2432 ± 1806	3699 ± 1043	6118 ± 2866	*F* (2, 56) = 16.5***	A** B***	

In Table 2a, *t*wo-tailed *t* tests for paired samples were used.

*SD* Standard deviation, *AD* Alzheimer’s disease, *n.s*. not significant

Α:Difference between Young adults and Older adults. Two-tailed *t* tests for independent samples were used

Β:Difference between Older adults and AD patients. Two-tailed *t* tests for independent samples were used

* *p* < 0.05. ** *p* < 0.01. *** *p*  < 0.001

### Surprise recognition phase

The 2 × 3 two-way repeated measures ANOVA with Gaze Direction as within-subjects factor and Group as between-subjects factor performed on the recognition rate revealed a main effect of group (*F*_(2,56)_ = 17.4, *p* < 0.0001; *η*^*2*^_*p*_ = 0.38). Planned comparisons revealed that young adults were significantly more accurate in recognizing old faces than healthy older participants (respectively, mean percentage of hits = 89 ± 8 Vs. 76 ± 11%, *t*_(38)_ = 3.98; *p* < 0.001, *d* > 2), who were more accurate than AD patients (mean percentage of Hits = 66 ± 15%; *t*_(37)_ = 2.25; *p* < 0.05, *d* > 2). There was no overall effect of gaze direction (*F*_(1,56)_ = 1.97 *p* < 0.16), or interaction between Gaze Direction and Group (*F*_(2,56)_ = 1.45; *p* < 0.24). However, as the literature reports a robust effect of direct gaze on memory for faces in young adults, we applied planned comparisons on the interaction between Gaze Direction and Group. They revealed that young adults recognized significantly more individuals initially displayed with direct than averted gaze (respectively, mean percentage of hits = 93 ± 9 Vs. 84 ± 13%, *t*_(19)_ = 2.71; *p* < 0.01, *d* = 0.61). However, this effect was absent in the other groups of participants (all *t* < 1; all *p* > 0.1) (Fig. 2b).

The 2 × 3 two-way repeated measures ANOVA with Gaze direction as within-subjects factor and Group as between-subjects factor performed on the RT for Hits revealed a main effect of Group on this variable (*F*_(2,56)_ = 16.49; *p* < 0.0001; *η*^*2*^_*p*_ = 0.37). Planned comparisons showed that, overall, AD participants were slower than healthy older adults (respectively, mean = 6118 ± 2866 vs. 3699 ± 1043 ms; *t*_(37)_ = 3.53; *p* < 0.001, *d* = 1.2) who were slower than young adults (mean = 2432 ± 1806 ms; *t*_(38)_ = 2,71; *p* < 0.01, *d* = 0.89) (Table 2b). No effects of Gaze direction, or interaction between Gaze Direction and Group, were found on RT’s for Hits (all *p* > 0.1) (Table 2a).

### Correlations between recognition rate and rating of likeability

**Fig. 3:**
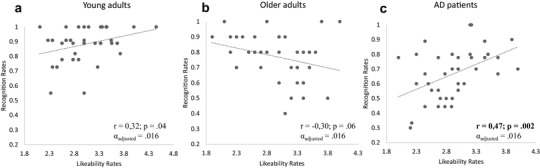
Correlation between mean ratings of likeability and mean recognition rate for each stimulus in **a** young adults, **b** healthy older adults and **c** AD patients

The correlation performed on mean ratings of Likeability and Recognition rates obtained for each face, independently of its gaze direction at the encoding phase, showed a strong positive correlation in AD patients only (*r* = 0,47, *p* = 0.002, *α*_adjusted_ = 0.016) (Fig. 3c). The more AD participants judged individuals as likeable, the more frequently these individuals were recognized in the recognition phase. This correlation was not observed in healthy older adults (*r* = − 0.30; *p* = 0.06, *α*_adjusted_ = 0.016) (Fig. 3b), or in young adults (*r* = 0.32; *p* = 0.04, *α*_adjusted_ = 0.016, Fig. 3a). The difference between the correlation coefficients obtained from AD patients and healthy older adults groups was statistically significant (*p* < 0.001). This was also the case when comparing the correlation coefficients obtained from young adults and healthy older adults groups (*p* < 0.01), while no difference was found between young adults and AD patients groups’ correlation coefficients (*p* > 0.1).

## Discussion

The present experiment tested the persistence of the eye contact effects in older adults and in AD patients. It has been shown that eye contact impacts normal cognition in several ways (Conty et al., 2016). Here, we focused on the impact of direct gaze on the modulation of others’ appraisal and memory of identity. Our main finding was that the effect of eye contact on other’s appraisal was preserved in normal ageing and in mild AD. By contrast, the effect of eye contact on memory for identity was not observed in both groups of older participants. Interestingly, the more AD patients judged the individuals likeable, the higher their recognition performances were. This suggests that AD patients implicitly allocated their encoding resources to most likeable faces.

### The evolution of the eye contact effects in older adults and in AD patients

The present data confirm the robust findings that direct gaze perception enhances memory for identity and favours positive appraisal of others in young adults (Conty & Grèzes, 2011; Kleinke, 1986; Kuzmanovic et al., 2009; Mason et al., 2004). Interestingly, the results revealed for the first time that the effect of direct gaze on other’s appraisal is preserved in normal ageing as well as until the mild stage of AD. Since effect sizes are similar across the groups, the intensity of this effect did not show any decline in our group of older participants (with or without AD). Moreover, this result confirmed indirectly older adults’ and AD patients’ ability to detect marked differences in gaze direction, in line with the findings of Slessor et al. (2008) and Bediou et al. (2009).

Not surprisingly, our results showed that, overall, healthy older adults were significantly slower and less accurate than young adults in recognizing faces. These effects can be related to the general weakening of encoding processing in normal ageing (for a review, see Buckner, 2004). The performances of AD patients decrease even more (when compared to healthy older adults), as encoding capacities become clearly pathological in mild AD (Ober, Koss, Friedland, & Delis, 1985). No key interaction between the variables “Group” and “Gaze direction” was found. Yet, planned comparisons revealed that direct gaze perception was associated with an increase of memory for identity in young adults, as it is also robustly reported in the literature (Conty & Grezes, 2011; Hood et al., 2003; Vuilleumier et al., 2005). Importantly, this association was not observed in older adults, nor in AD patients. The effect of direct gaze on memory would decline with ageing, along with cognitive efficiency and social skills (Slessor et al., 2008; Henry, Phillips, Ruffman, & Bailey, 2013). Thus, our result thus suggests that processing related to direct gaze does not allow compensation of the encoding decline of faces in healthy older adults and AD patients.

Recently, Conty et al. (2016) argued that eye contact effects rely on a unique mechanism of self-referential i.e. a heightened involvement of the self that modulates incoming information processing. This implies that the eye contact effects evolve together in normal ageing. The present results contradict this view and suggest that each eye contact effect depends on specific mechanisms, at least partly. In particular, the memory effect of direct gaze could require intact encoding capacities to emerge. Future studies involving older participants and a more sensitive neuropsychological test measuring of encoding abilities are needed to clarify this issue. Still, the proposal that eye contact effects also rely on self-referential mechanisms cannot be rejected and opens the possibility that the memory effect of eye contact for identity would emerge in older adults when increasing the relevance of the perceived faces (see Northoff et al., 2006 for the link between self-relevance and self-referential processing).

It is possible, for example, that photographs of faces shown on a computer screen were less relevant to process for older adults than for young adults. While such a method is classically used to investigate the effect of direct gaze in memory for faces, it has been proven to be efficient in infants and young adults only. In the same line, an own-age bias in attention and memory for faces has been reported for both young and older adults, caused by the increased personal and social relevance of own-age compared to other-age faces (for a review see Rhodes & Anastasi, 2012). The age of each individual presented in our stimuli ranges from 20 to 60 years. This may have favored an own-age bias in young adults but not in older adults (with or without AD) since the mean age for both groups was over 75 years. This hypothesis is weakened by recent results reporting an absence of own-age bias in older adults in a gaze following task (Slessor, 2010). Moreover, the choice of our stimuli enhances the ecological validity of our study which addresses the question of whether the eye contact effects actually occur in older adults or AD patients on a daily basis, when one is likely to meet people of all ages. Still, it is possible that improving the relevance of the faces (in particular, using own-age faces or by instructing the participant to memorise the faces—see below—) favours self-referential processing, allowing the memory effect of eye contact for identity to emerge in older adults.

It is also noteworthy that in the present task, participants were explicitly asked to judge the likeability of the face, while they were then tested on their implicit face encoding processes. One could argue that the memory effect of eye contact for identity would emerge in older adults if participants are explicitly asked to encode the faces. We have chosen to use an incidental encoding memory task for two reasons. First, the option to explicitly ask to “use memory” may trigger anxiety in AD patients. As a matter of fact, despite the quite common presence of anosognosia in AD patients (i.e. the lack of awareness of neurological deficits; American Psychiatric Association 2013), they may still remain partially aware and concerned about their memory impairments as healthy older adults (Feher, Mahurin, Inbody, Crook, & Pirozzolo, 1991; Starkstein, Jorge, Mizrahi, & Robinson, 2006). Moreover, the incidental encoding task is assumed to possess a more ecological validity as it resembles the formation of memory during everyday life, where instructions to remember certain stimuli are rarely present. Future studies should investigate the effect of eye contact on memory for identity in older adults, using explicit instructions.

One may note that, under certain conditions, direct gaze may be considered as an aversive stimulus, leading humans to avoid eye contact in social situations (e.g. Gallup, Chong, & Couzin, 2012). However, the existing literature suggests that the aversive nature of direct gaze depends on the gaze duration. The preferred direct gaze duration is around 3–4 s (Argyle & Cook, 1976; Argyle & Ingham, 1972; Binetti et al., 2016), whilst the “uncomfortable feeling” of being gazed seems to increase after that (Binetti et al., 2016). Moreover, in our study, participants were exposed to direct gazes for the time they needed to perform the rating of likeability. The mean duration of direct gaze exposure exceeded 4 s only in the AD group. It is possible that AD patients’ exposition times to our stimuli with direct gaze made them actually feel uncomfortable and thus limiting the effect of eye contact on memory. However, this possibility seems unlikely, since none of the AD participants has reported such discomfort.

### The correlation between likeability judgements and memory for faces

We further explored the link between ratings of likeability and recognition rate for faces, as likeability judgement may play a mediating role in face recognition (Bruce & McDonald, 1993; Mueller, Heesacker, & Ross, 1984). Interestingly, our results revealed that AD patients encode face’s identity as a function of their judgement of likeability. Such correlation is not observed in young adults (not surprisingly, since their performance on recognition showed a ceiling effect) or in healthy older adults.

The few existing studies related to the effect of face likeability on memory show mixed results. One study has shown that unlikeable faces were better remembered compared to likeable faces (Mueller et al., 1984). By contrast, another study has reported worse memory for unlikeable compared to likeable faces (Bruce & McDonald, 1993). More recently, Lin, Lendry, and Ebner (2015) investigated the possible mediating role of face likeability on the relationship between face attractiveness and memory. Face likeability and face attractiveness are two concepts partially overlapping but not identical [see, for example, Ebner, (2008)]. Interestingly, the author also explored the age-related change on such dynamics. Their results showed a memory-enhancing effect of face attractiveness in young—but not in older—participants, which was partially mediated by face likeability. To explain their results, the authors suggested that face attractiveness and face likeability might influence memory as a function of age-related changes in primary social goals. For example, a temporary life-goal typically associated with young age, such as developing a romantic relationship, may explain the better encoding of likeable/attractive faces in young—but not in older—adults. This may offer an explanation for the lack of correlation between likeability judgement and recognition rate in our older adults control group.

However, our AD group of participants did encode the faces as a function of their likeability. Very little is known about social motivation in Alzheimer disease or even, more generally, in dementia. We know that people with dementia are still engaged and are willing to engage themselves in social interactions (Mabire, Gay, Vrignaud, Garitte, & Vernooij-Dassen, 2016; Rousseaux et al., 2010) despite their difficulties in communicating with others (Parsons-suhl et al., 2008; Snyder, 2001). However, as we have already pointed out, AD patients are partially aware and concerned about their memory impairments and the impoverishment of their social relations. The fact that they encoded face’s identity as a function of their judgement of likeability may actually be the result of an implicit strategy, which they develop to overcome their memory impairments when it comes to deal with social situations, e.g. when making new acquaintances. This should be subtended by a form of emotional memory enhancement, i.e. the tendency of emotional content to be better-remembered than non-emotional content, which is reported as being intact in people with AD (for a review, see Broster, Blonder, & Jiang, 2012). The present result may, thus, reflect an “affective memory” for faces: likeable faces could trigger an emotional encoding of face in AD patients leading to a better memory trace.

## Conclusion

In conclusion, our study replicated previous findings concerning the existence of two eye contact effects in young population, namely positive appraisal of others and memory for other’s identity. Importantly, we provide the first direct behavioural evidence that the eye contact effect on other’s appraisal is preserved in normal ageing population as well as in AD patients. By contrast, our findings suggest that the effect of eye contact on memory for faces declines with age. However, increasing the relevance of the faces and/or instructing explicitly the participants to encode the faces, may lead the effect to emerge in older adults.

Finally, we showed that AD patients encode faces as a function of their likeability. This result suggests that despite the lack of direct gaze effect on face recognition in our study, memory performances for faces could still be improved in AD patients by taking advantage of the positive impact of direct gaze on social judgement. Interestingly, this finding can be easily translated in clinical practice by encouraging professional caregivers to maximize eye contact behaviours with AD patients to increase the chances to easily establish a fulfilling patient–carer relationship.
